# Supplementary material for: Fast and accurate Ab Initio Protein structure prediction using deep learning potentials
Source: PLoS Comput Biol. 2022 Sep 16;18(9):e1010539. doi: 10.1371/journal.pcbi.1010539 (PMC9518900; doi:10.1371/journal.pcbi.1010539)
Supplement: S1 Table — (PDF) [file pcbi.1010539.s001.pdf]

Table S1: Impact of the different components of the DeepFold energy function on the structure modeling accuracy in terms of the average TM-score and the percent of correctly folded models (TM-scores $\geq$0.5) for the 221 benchmark proteins. The *p*-values were calculated using paired, two-sided Student's t-tests.

Energy Function	TM-score (*p*-value)	Correct Folds
GE	0.184 (8.4E-127)	0.0%
GE+Cont	0.263 (1.3E-118)	1.8%
GE+Cont+Dist	0.677 (1.9E-14)	76.0%
GE+Cont+Dist+Orien	**0.751 (*)**	**92.3%**
