# Supplementary material for: Fast and accurate Ab Initio Protein structure prediction using deep learning potentials
Source: PLoS Comput Biol. 2022 Sep 16;18(9):e1010539. doi: 10.1371/journal.pcbi.1010539 (PMC9518900; doi:10.1371/journal.pcbi.1010539)
Supplement: S2 Table — (PDF) [file pcbi.1010539.s002.pdf]

Table S2: Mean absolute error (MAE) between the distance maps predicted by DeepPotential and the distance maps of the 3D models built without (GE+Cont+Dist) and with (GE+Cont+Dist+Orien) inter-residue orientations. Here, the top *n*L* long-range distance restraints were sorted by their DeepPotential confidence scores. The *p*-values were calculated using paired, two-sided Student's t-tests.

**Method**	**L/2 (*p*-value)**	**L (*p*-value)**	**2L (*p*-value)**	**5L (*p*-value)**	**10L (*p*-value)**
GE+Cont+Dist	0.692 (2.3E-09)	0.707 (5.9E-10)	0.738 (1.0E-10)	0.857 (9.1E-10)	1.074 (1.5E-06)
GE+Cont+Dist+Orien	**0.562 (*)**	**0.577 (*)**	**0.606 (*)**	**0.704 (*)**	**0.887 (*)**
