# Supplementary material for: Fast and accurate Ab Initio Protein structure prediction using deep learning potentials
Source: PLoS Comput Biol. 2022 Sep 16;18(9):e1010539. doi: 10.1371/journal.pcbi.1010539 (PMC9518900; doi:10.1371/journal.pcbi.1010539)
Supplement: S3 Table — (PDF) [file pcbi.1010539.s003.pdf]

Table S3: DeepFold results on the 38 β-proteins in the test set with and without orientation restraints in terms of the average TM-score/RMSD and the percent of correctly folded models (TM-scores ≥0.5) for the 221 benchmark proteins. The *p*-values were calculated using paired, two-sided Student's t-tests.

Method	TM-score (*p*-value)	RMSD (*p*-value)	Correct Folds
GE+Cont+Dist	0.590 (1.5E-04)	8.42 (3.4E-04)	60.5%
GE+Cont+Dist+Orien	**0.706 (*)**	**6.12 (*)**	**86.8%**
