# Supplementary material for: Fast and accurate Ab Initio Protein structure prediction using deep learning potentials
Source: PLoS Comput Biol. 2022 Sep 16;18(9):e1010539. doi: 10.1371/journal.pcbi.1010539 (PMC9518900; doi:10.1371/journal.pcbi.1010539)
Supplement: S4 Table — (PDF) [file pcbi.1010539.s004.pdf]

Table S4: Impact of the general energy (GE) function on DeepFold's modeling performance. Specifically, the table presents the effect of the GE on the secondary structure SOV score, number of Ramachandran outliers, the MolProbity clash score, and the total MolProbity score on the overall dataset and those targets with poor physical model quality.

**Target Type (# of Proteins)**	**DeepFold Energy Function**	**SS SOV**	**Rama Outliers**	**Clash Score**	**MP-score**
All Targets (221)	w/o General Energy	79.68%	6.52	3.61	1.735
	with General Energy	**79.71%**	**5.92**	**3.13**	**1.692**
MP-score <50th Percentile (16)	w/o General Energy	58.41%	13.00	17.54	2.882
	with General Energy	**61.44%**	**9.81**	**8.58**	**2.308**
