# Supplementary material for: Fast and accurate Ab Initio Protein structure prediction using deep learning potentials
Source: PLoS Comput Biol. 2022 Sep 16;18(9):e1010539. doi: 10.1371/journal.pcbi.1010539 (PMC9518900; doi:10.1371/journal.pcbi.1010539)
Supplement: S5 Table — (PDF) [file pcbi.1010539.s005.pdf]

Table S5: Results on the 221 benchmark proteins in terms of the median TM-scores and RMSDs, where the *p*-values were calculated using two-sided, non-parametric Wilcoxon signed rank tests.

**Method**	**Median TM-score (*p*-value)**	**Median RMSD (*p*-value)**
I-TASSER	0.357 (3.1E-37)	14.10 (1.2E-35)
C-I-TASSER	0.607 (1.9E-35)	7.00 (6.7E-27)
DMPfold	0.710 (2.0E-34)	5.96 (4.4E-23)
trRosetta	0.749 (1.6E-26)	4.59 (3.3E-16)
DeepFold	**0.800 (*)**	**3.94 (*)**
