# Supplementary material for: Fast and accurate Ab Initio Protein structure prediction using deep learning potentials
Source: PLoS Comput Biol. 2022 Sep 16;18(9):e1010539. doi: 10.1371/journal.pcbi.1010539 (PMC9518900; doi:10.1371/journal.pcbi.1010539)
Supplement: S6 Table — (PDF) [file pcbi.1010539.s006.pdf]

Table S6: MAEs of the top *n*L* long-range distances by different distance predictors on the 221 test proteins. The *p*-values were calculated using paired, two-sided Student's t-tests between the DeepPotential results and the control methods.

**Method**	**L/2 (*p*-value)**	**L (*p*-value)**	**2L (*p*-value)**	**5L (*p*-value)**	**10L (*p*-value)**
DeepPotential	**0.974 (*)**	**1.018 (*)**	**1.090 (*)**	**1.302 (*)**	**1.613 (*)**
trRosetta	1.050 (4.9E-02)	1.154 (5.9E-04)	1.328 (2.8E-06)	1.730 (2.0E-07)	2.241 (1.4E-11)
DMPfold	1.779 (1.4E-15)	1.930 (7.6E-22)	2.184 (7.5E-28)	2.695 (1.6E-33)	3.488 (1.1E-41)
