# Supplementary material for: Fast and accurate Ab Initio Protein structure prediction using deep learning potentials
Source: PLoS Comput Biol. 2022 Sep 16;18(9):e1010539. doi: 10.1371/journal.pcbi.1010539 (PMC9518900; doi:10.1371/journal.pcbi.1010539)
Supplement: S7 Table — (PDF) [file pcbi.1010539.s007.pdf]

Table S7: Modeling results for trRosetta using DeepPotential's spatial restraints vs DeepFold, where the *p*-value for the mean TM-score was calculated using a paired, two-sided Student's t-test, while the *p*-value for the median TM-score were calculated using a two-sided, non-parametric Wilcoxon signed rank test.

**Method**	**Mean TM-score (*p*-value)**	**Median TM-score (*p*-value)**	**Correct Folds**
trRosetta+DeepPotential	0.735 (3.9E-09)	0.787 (4.2E-13)	90.5%
DeepFold	**0.751 (*)**	**0.800 (*)**	**92.3%**
