# Supplementary material for: Fast and accurate Ab Initio Protein structure prediction using deep learning potentials
Source: PLoS Comput Biol. 2022 Sep 16;18(9):e1010539. doi: 10.1371/journal.pcbi.1010539 (PMC9518900; doi:10.1371/journal.pcbi.1010539)
Supplement: S8 Table — (PDF) [file pcbi.1010539.s008.pdf]

Table S8: Results on the 90 proteins that were non-redundant to the training set of DeepPotential. For the mean TM-scores, the *p*-values were calculated using paired, two-sided Student's t-tests, while the *p*-values for the median TM-scores were calculated using two-sided, non-parametric Wilcoxon signed rank tests.

**Method**	**Mean TM-score (*p*-value)**	**Median TM-score (*p*-value)**	**Correct Folds***	**$TM_{DeepFold} > TM_{Method}$‡**
I-TASSER	0.384 (8.5E-31)	0.346 (4.7E-16)	28.9%	93.3%
C-I-TASSER	0.580 (3.0E-20)	0.605 (7.7E-15)	66.7%	93.3%
DMPfold	0.643 (6.4E-16)	0.700 (2.6E-15)	76.7%	90.0%
trRosetta	0.688 (7.5E-09)	0.719 (2.6E-09)	87.8%	83.3%
DeepFold	**0.730 (*)**	**0.778 (*)**	**90.0%**	**-**

* This column represents the percent of proteins with TM-scores ≥0.5.

‡ This column indicates the percent of test proteins for which DeepFold generated a model with a higher TM-score than the control method.
