# Supplementary material for: Fast and accurate Ab Initio Protein structure prediction using deep learning potentials
Source: PLoS Comput Biol. 2022 Sep 16;18(9):e1010539. doi: 10.1371/journal.pcbi.1010539 (PMC9518900; doi:10.1371/journal.pcbi.1010539)
Supplement: S9 Table — (PDF) [file pcbi.1010539.s009.pdf]

Table S9: Modeling results for DeepFold and AlphaFold on the 31 CASP13 FM targets that the AlphaFold team submitted models for, where the *p*-values for the mean/median TM-scores were calculated using a paired, two-sided Student's t-tests and a two-sided Wilcoxon signed rank test, respectively.

**Method**	**Mean TM-score (*p*-value)**	**Median TM-score (*p*-value)**	**Correct Folds**
AlphaFold	0.589 (0.025)	0.641 (0.044)	64.5%
DeepFold	**0.636**	**0.672**	**80.6%**
