# Supplementary material for: Fast and accurate Ab Initio Protein structure prediction using deep learning potentials
Source: PLoS Comput Biol. 2022 Sep 16;18(9):e1010539. doi: 10.1371/journal.pcbi.1010539 (PMC9518900; doi:10.1371/journal.pcbi.1010539)
Supplement: S10 Table — (PDF) [file pcbi.1010539.s010.pdf]

Table S10: Modeling results of DeepFold using the DeepPotential restraints vs RosettaFold/AlphaFold2 on the 221 test proteins. For the mean TM-scores, the *p*-values were calculated using paired, two-sided Student's t-tests, while the *p*-values for the median TM-scores were calculated using two-sided, non-parametric Wilcoxon signed rank tests.

Method	Mean TM-score (*p*-value)	Median TM-score (*p*-value)	Correct Folds*
RosettaFold (End-to-End)	0.812 (3.6E-10)	0.872 (3.8E-12)	93.7%
RosettaFold (Pyrosetta)	0.838 (8.0E-22)	0.884 (1.5E-27)	**95.5%**
AlphaFold2	**0.903 (1.4E-49)**	**0.951 (4.1E-35)**	95.0%
DeepFold	0.751	0.800	92.3%

* This column represents the percent of proteins with TM-scores ≥0.5.
