# Supplementary material for: Fast and accurate Ab Initio Protein structure prediction using deep learning potentials
Source: PLoS Comput Biol. 2022 Sep 16;18(9):e1010539. doi: 10.1371/journal.pcbi.1010539 (PMC9518900; doi:10.1371/journal.pcbi.1010539)
Supplement: S11 Table — (PDF) [file pcbi.1010539.s011.pdf]

Table S11: Modeling results of DeepFold using the combined RosettaFold/DeepPotential restraints vs RosettaFold/AlphaFold2 on the 221 test proteins. For the mean TM-scores, the *p*-values were calculated using paired, two-sided Student's t-tests, while the *p*-values for the median TM-scores were calculated using two-sided, non-parametric Wilcoxon signed rank tests.

**Method**	**Mean TM-score (*p*-value)**	**Median TM-score (*p*-value)**	**Correct Folds***
RosettaFold (End-to-End)	0.812 (2.4E-11)	0.872 (1.2E-11)	14.3%
RosettaFold (Pyrosetta)	0.838 (1.2E-02)	0.884 (8.9E-02)	95.5%
AlphaFold2	**0.903 (4.1E-11)**	**0.951 (6.3E-25)**	95.0%
DeepFold	0.844	0.889	**96.4%**

* This column represents the percent of proteins with TM-scores $\geq$0.5.
