# Supplementary material for: Fast and accurate Ab Initio Protein structure prediction using deep learning potentials
Source: PLoS Comput Biol. 2022 Sep 16;18(9):e1010539. doi: 10.1371/journal.pcbi.1010539 (PMC9518900; doi:10.1371/journal.pcbi.1010539)
Supplement: S12 Table — (PDF) [file pcbi.1010539.s012.pdf]

Table S12: Selection of the first well width (d_b) in the contact potential for various protein lengths (L in AA).

	L<100	L in [100,120]	L in [120,200]	L>200
Start of 1st well	8	8	8	8
Width of 1st well, d_b	6	8	10	12
End of 1st & start of 2nd well, $D=(8+d_b)$	14	16	18	20
Width of 2nd well, $(80-D)$	66	64	62	60
