# Supplementary figures and images for: Fast and accurate Ab Initio Protein structure prediction using deep learning potentials
Source: PLoS Comput Biol. 2022 Sep 16;18(9):e1010539. doi: 10.1371/journal.pcbi.1010539 (PMC9518900; doi:10.1371/journal.pcbi.1010539)

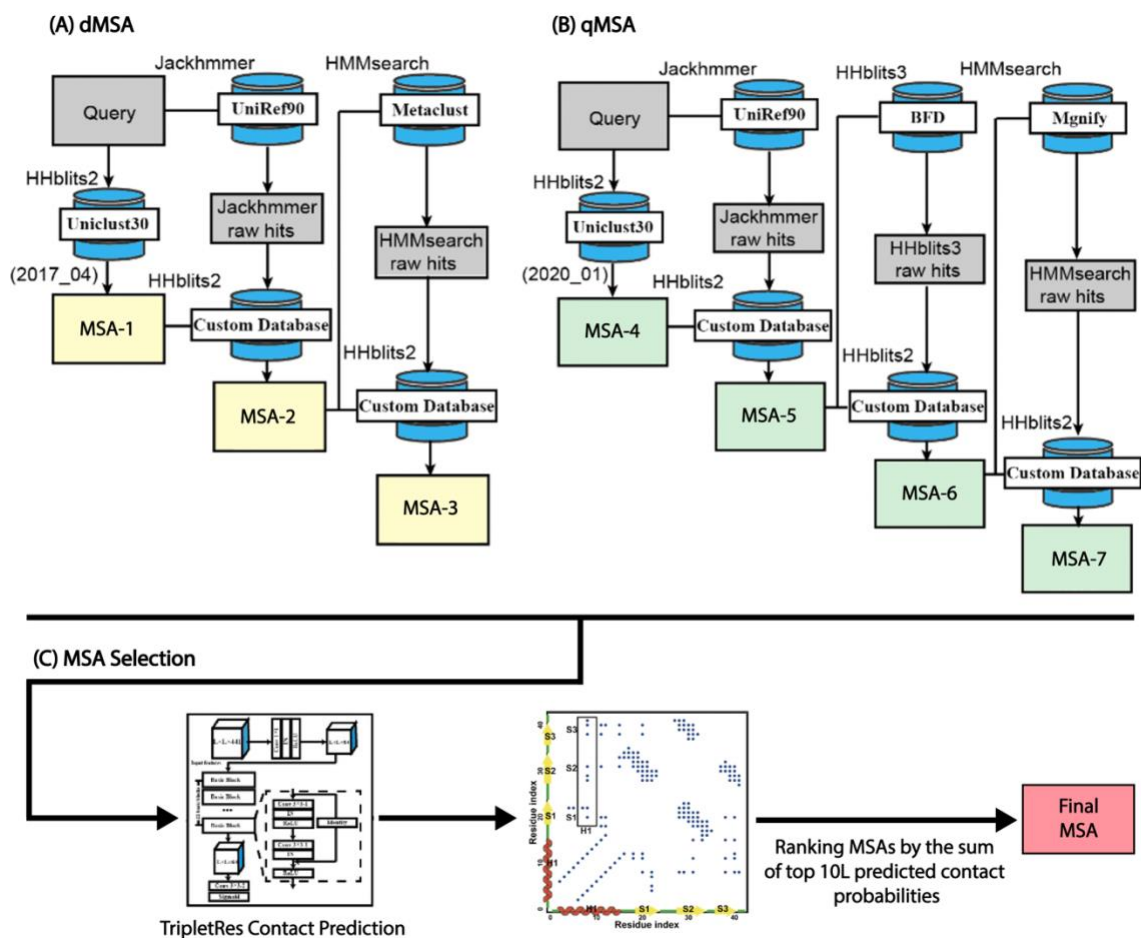

**Figure S1.** DeepMSA2 pipeline, which contains three major steps: (A) dMSA, (B) qMSA, and (C) MSA selection.

Supplement: S1 Fig — DeepMSA2 pipeline, which contains three approaches, (A) dMSA, (B) qMSA, and (C) MSA selection. (PDF) [file pcbi.1010539.s013.pdf]
