# Supplementary material for: Fast and accurate Ab Initio Protein structure prediction using deep learning potentials
Source: PLoS Comput Biol. 2022 Sep 16;18(9):e1010539. doi: 10.1371/journal.pcbi.1010539 (PMC9518900; doi:10.1371/journal.pcbi.1010539)
Supplement: S2 Fig — (PDF) [file pcbi.1010539.s014.pdf]

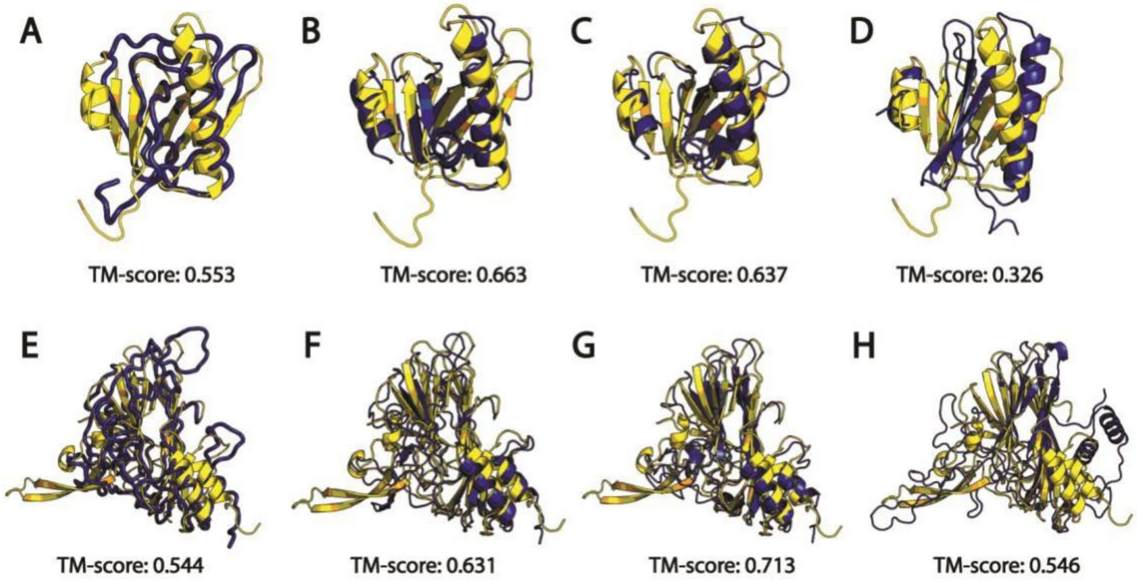

Figure S2. Case study from two targets, d1ltrd (A-D) and d1nova (E-H), for which I-TASSER/C- I-TASSER outperformed DeepFold. A) LOMETS template (blue) superposed with the native structure for d1ltrd (yellow); B) I-TASSER model (blue) superposed with the native structure (yellow); C) C-I-TASSER model (blue) superposed with the native structure (yellow); D) DeepFold model (blue) superposed with the native structure (yellow); E) LOMETS template (blue) superposed with the native structure for d1nova (yellow); F) I-TASSER model (blue) superposed with the native structure (yellow); G) C-I-TASSER model (blue) superposed with the native structure (yellow); H) DeepFold model (blue) superposed with the native structure (yellow).
