# Supplementary material for: Fast and accurate Ab Initio Protein structure prediction using deep learning potentials
Source: PLoS Comput Biol. 2022 Sep 16;18(9):e1010539. doi: 10.1371/journal.pcbi.1010539 (PMC9518900; doi:10.1371/journal.pcbi.1010539)
Supplement: S3 Fig — (PDF) [file pcbi.1010539.s015.pdf]

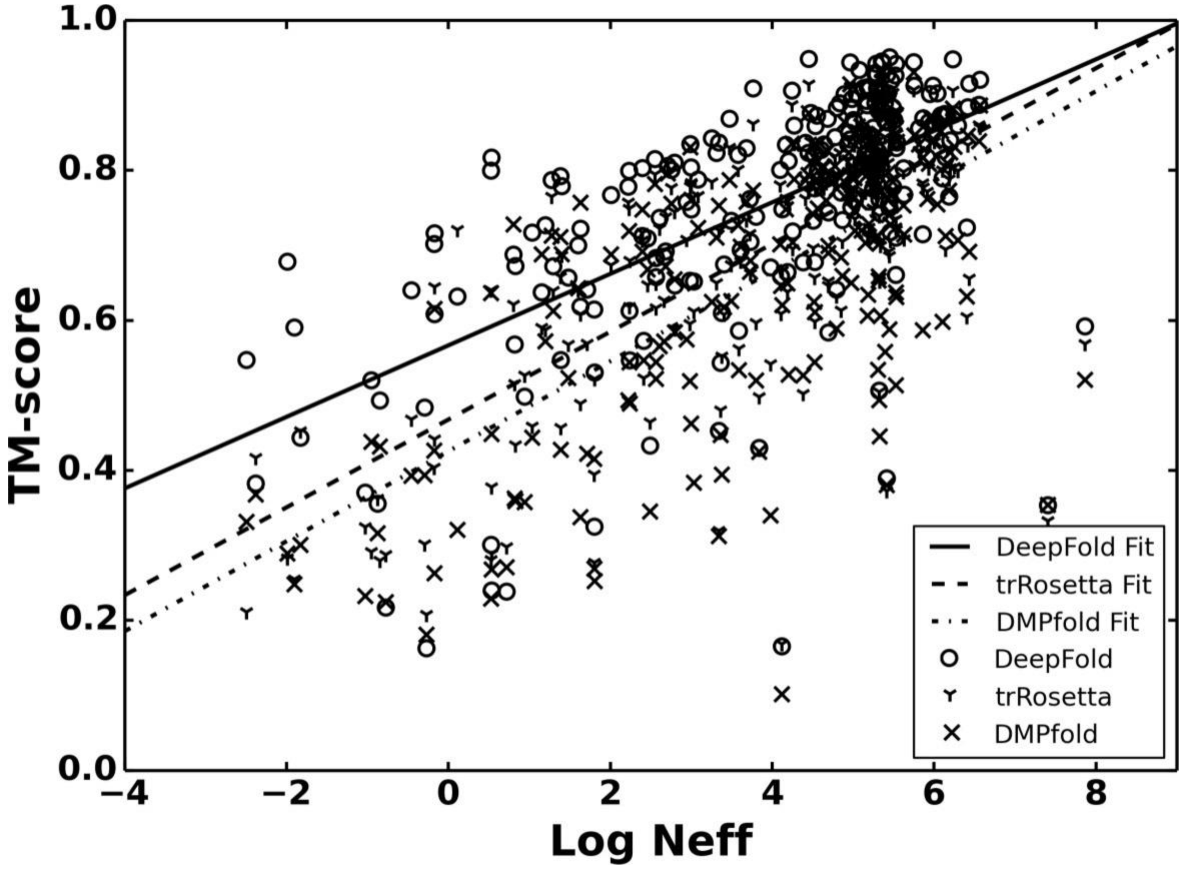

Figure S3. Model TM-score vs. the logarithm of the MSA Neff value for DeepFold, trRosetta, and DMPfold, where the fitted models were obtained by linear regression with Pearson's Correlation Coefficients of 0.615, 0.712, and 0.675 for DeepFold, trRosetta, and DMPfold, respectively.
