# Supplementary material for: Fast and accurate Ab Initio Protein structure prediction using deep learning potentials
Source: PLoS Comput Biol. 2022 Sep 16;18(9):e1010539. doi: 10.1371/journal.pcbi.1010539 (PMC9518900; doi:10.1371/journal.pcbi.1010539)
Supplement: S4 Fig — (PDF) [file pcbi.1010539.s016.pdf]

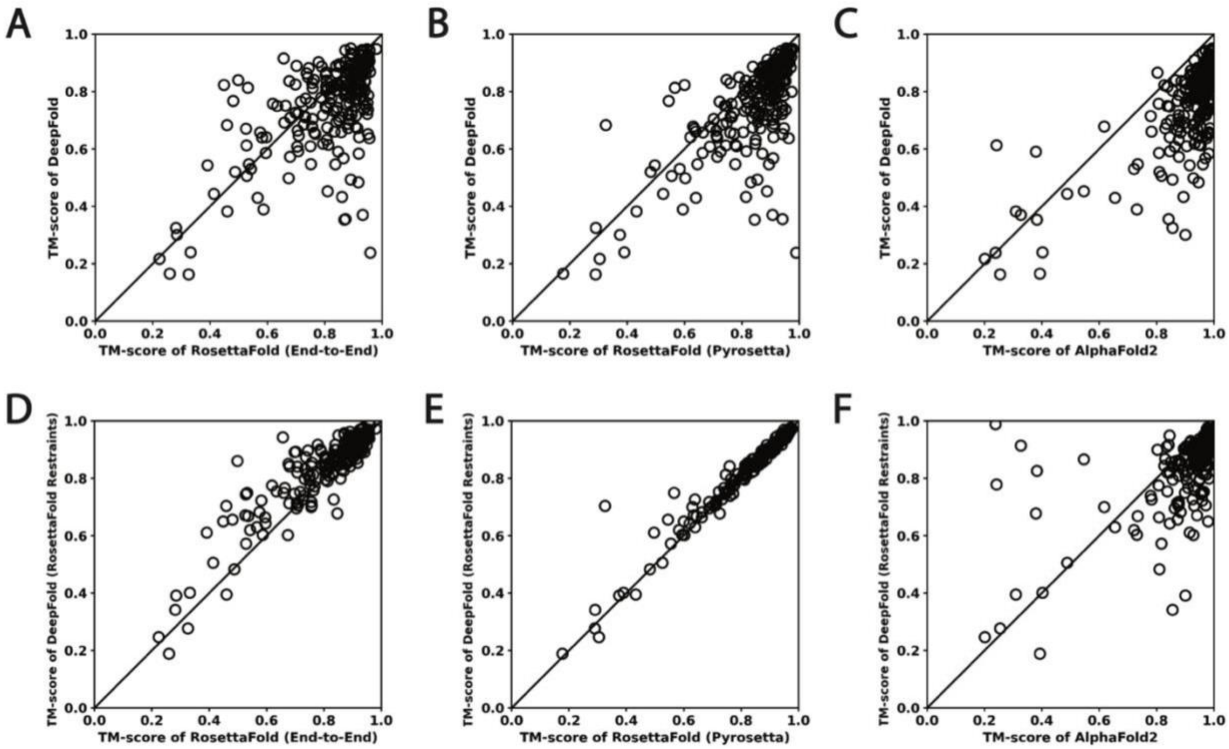

Figure S4. Head-to-head TM-score comparisons between DeepFold using the restraints from DeepPotential (A-C) or the combined restraints from RosettaFold and DeepPotential (D-F) with other protein structure prediction methods on the 221 Hard benchmark proteins: A/D) RosettaFold (End-to-End); B/E) RosettaFold (Pyrosetta); C/F) AlphaFold2.
