# Supplementary material for: Fast and accurate Ab Initio Protein structure prediction using deep learning potentials
Source: PLoS Comput Biol. 2022 Sep 16;18(9):e1010539. doi: 10.1371/journal.pcbi.1010539 (PMC9518900; doi:10.1371/journal.pcbi.1010539)
Supplement: S5 Fig — (PDF) [file pcbi.1010539.s017.pdf]

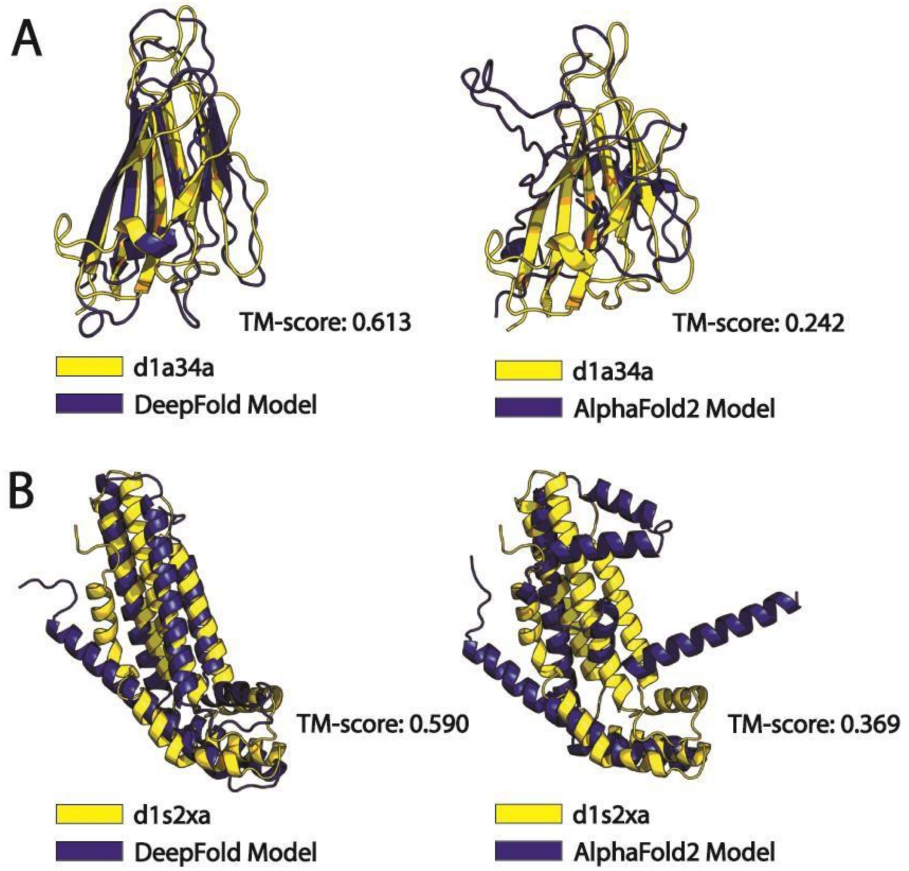

Figure S5. Case study from two proteins (d1a34a and d1s2xa) for which DeepFold significantly outperformed AlphaFold2. The DeepFold/AlphaFold2 models are shown in blue superposed with the native structures in yellow.
