# Supplementary material for: Fast and accurate Ab Initio Protein structure prediction using deep learning potentials
Source: PLoS Comput Biol. 2022 Sep 16;18(9):e1010539. doi: 10.1371/journal.pcbi.1010539 (PMC9518900; doi:10.1371/journal.pcbi.1010539)
Supplement: S6 Fig — (PDF) [file pcbi.1010539.s018.pdf]

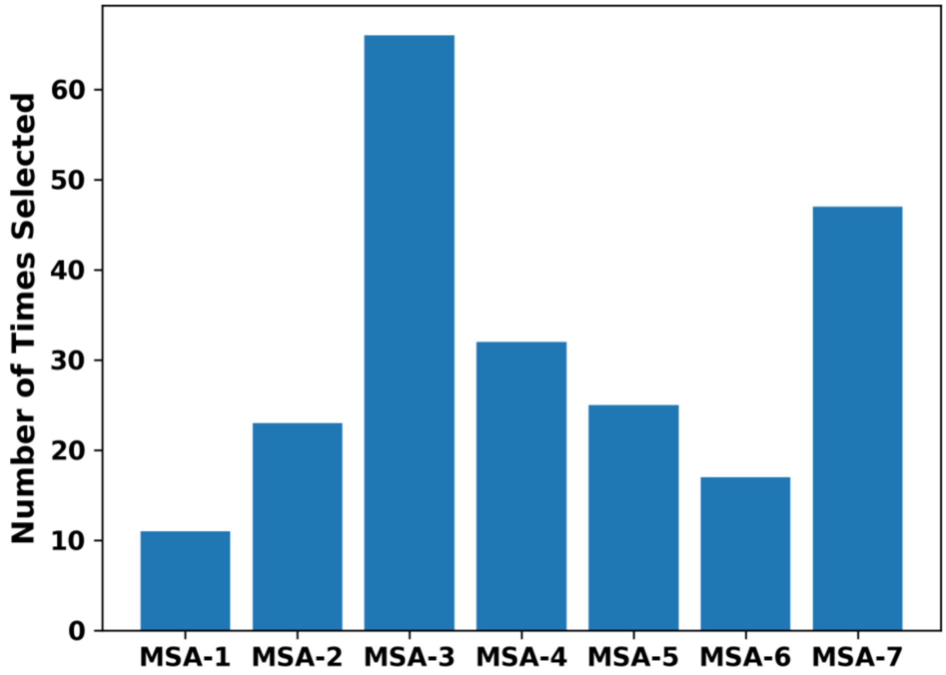

Figure S6. Histogram distribution of the number of times each of the 7 MSAs were selected by DeepMSA2 for the 221 benchmark targets. The MSA numbers correspond to those depicted in S1 Fig.
