# Supplementary material for: Fast and accurate Ab Initio Protein structure prediction using deep learning potentials
Source: PLoS Comput Biol. 2022 Sep 16;18(9):e1010539. doi: 10.1371/journal.pcbi.1010539 (PMC9518900; doi:10.1371/journal.pcbi.1010539)
Supplement: S7 Fig — (PDF) [file pcbi.1010539.s019.pdf]

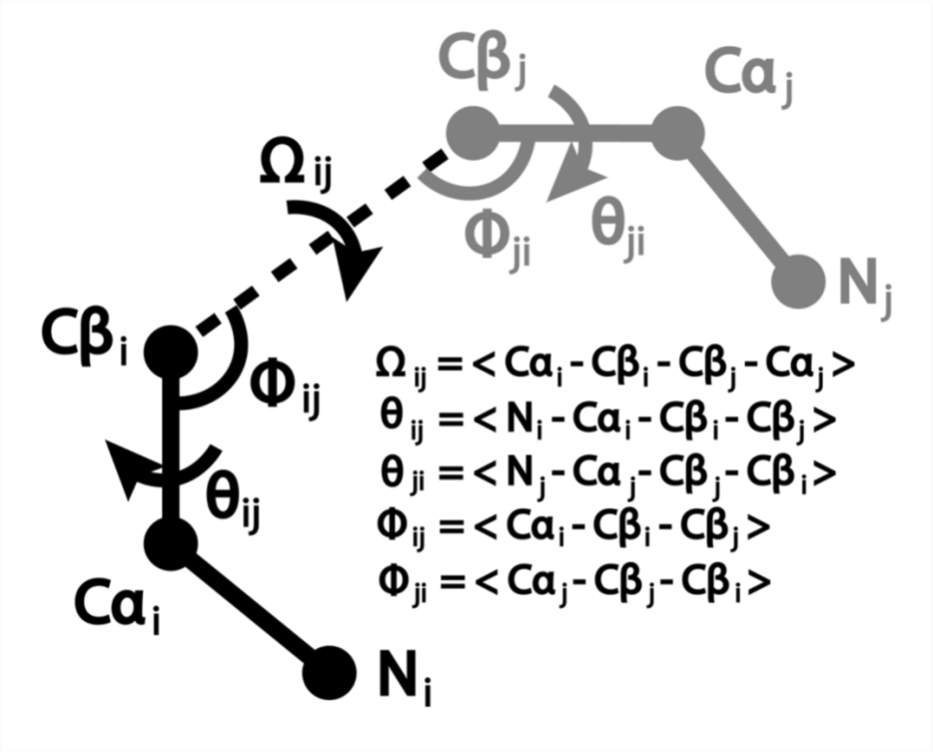

Figure S7. Definition of the inter-residue orientations predicted by DeepPotential, where Ω and θ are inter-residue torsion angles formed by the four indicated atoms and φ is an inter-residue angle formed by three atoms.
