# Supplementary material for: Fast and accurate Ab Initio Protein structure prediction using deep learning potentials
Source: PLoS Comput Biol. 2022 Sep 16;18(9):e1010539. doi: 10.1371/journal.pcbi.1010539 (PMC9518900; doi:10.1371/journal.pcbi.1010539)
Supplement: S8 Fig — (PDF) [file pcbi.1010539.s020.pdf]

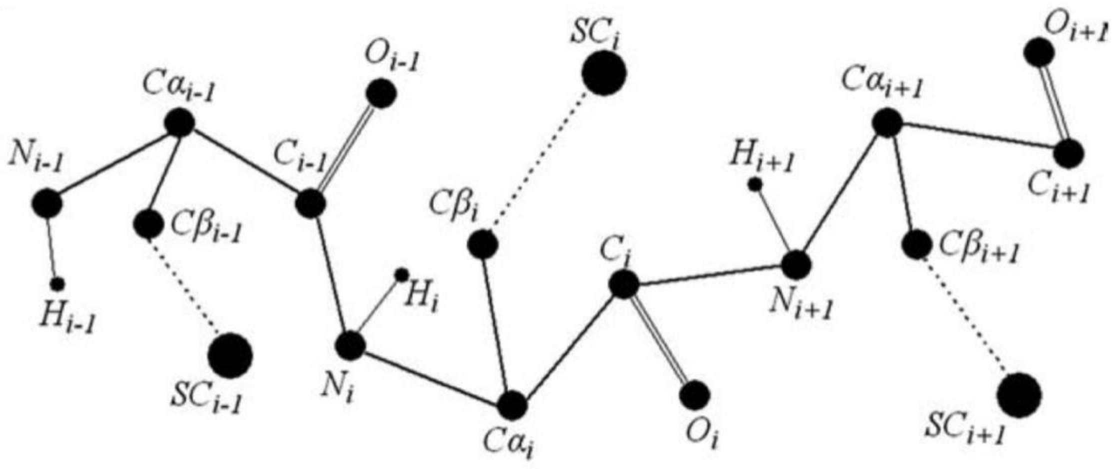

Figure S8. Depiction of the reduced model used to represent protein conformations during the DeepFold folding simulations, including the backbone atoms (N, H, Cα, C, and O) as well as the Cβ atoms and side-chain centers of mass for each amino acid type.
