# Supplementary material for: Fast and accurate Ab Initio Protein structure prediction using deep learning potentials
Source: PLoS Comput Biol. 2022 Sep 16;18(9):e1010539. doi: 10.1371/journal.pcbi.1010539 (PMC9518900; doi:10.1371/journal.pcbi.1010539)
Supplement: S1 Text — (PDF) [file pcbi.1010539.s021.pdf]

Text S1: Calculation of the MSA Neff value.

In order to quantify the quality of an MSA, we define the number of effective sequences (Neff) as follows:

$$Neff = \frac{1}{\sqrt{L}} \sum_{n=1}^{N} \frac{1}{1 + \sum_{m=1, m \neq n}^{N} I[S_{m,n} \geq 0.8]}$$

where L is the length of a query protein, N is the number of sequences in the MSA, $S_{m,n}$ is the sequence identity between the *m*-th and *n*-th sequences, and $I[\ ]$ represents the Iverson bracket, which means $I\left[S_{m,n \geq 0.8}\right] = 1$ if $S_{m,n} \geq 0.8$ or 0 otherwise.
