# Supplementary material for: Fast and accurate Ab Initio Protein structure prediction using deep learning potentials
Source: PLoS Comput Biol. 2022 Sep 16;18(9):e1010539. doi: 10.1371/journal.pcbi.1010539 (PMC9518900; doi:10.1371/journal.pcbi.1010539)
Supplement: S2 Text — (PDF) [file pcbi.1010539.s022.pdf]

Text S2: DeepFold energy function.

The energy function used to guide the DeepFold simulations is a combination of 10 energy terms:

$$E_{DeepFold} = (E_{C\beta dist} + E_{C\alpha dist} + E_{C\beta cont} + E_{C\alpha cont} + E_{\Omega} + E_{\theta} + E_{\varphi}) + (E_{hb} + E_{vdw} + E_{tor}) \quad (1)$$

where $E_{C\beta dist}$, $E_{C\alpha dist}$, $E_{C\beta cont}$, and $E_{C\alpha cont}$ are the predicted Cβ–Cβ distances, Cα–Cα distances, Cβ–Cβ contacts, and Cα–Cα contacts generated by DeepPotential; E_{Ω}, E_{θ}, and E_{φ} are the predicted inter-residue orientations by DeepPotential as defined in Fig. S7; and E_{hb}, E_{vdw}, and E_{tor} are the hydrogen bonding, van der Waals and backbone torsion angle potentials. All of the energy terms are based on pairwise interactions between residues *i* and *j* in a protein molecule, with the exception of E_{tor}, which is a single-body potential. Thus, the cumulative terms are derived from the summation over all residue pairs *i* and *j* as follows:

$$E_{C\beta dist} = \sum_{i,j} w_1 E_{d_{ij}}(i,j) \quad (2)$$

$$E_{C\alpha dist} = \sum_{i,j} w_2 E_{d_{ij}}(i,j) \quad (3)$$

$$E_{C\beta cont} = \sum_{i,j} w_3 E_{con_{ij}}(i,j) \quad (4)$$

$$E_{C\alpha cont} = \sum_{i,j} w_3 E_{con_{ij}}(i,j) \quad (5)$$

$$E_{\Omega} = \sum_{i,j} w_4 E_{\Omega_{ij}}(i,j) \quad (6)$$

$$E_{\theta} = \sum_{i,j} w_5 E_{\theta_{ij}}(i,j) + \sum_{j,i} w_5 E_{\theta_{ji}}(j,i) \quad (7)$$

$$E_{\varphi} = \sum_{i,j} w_6 E_{\varphi_{ij}}(i,j) + \sum_{j,i} w_6 E_{\varphi_{ji}}(j,i) \quad (8)$$

$$E_{\mathrm{hb}} = \sum_{i,j} w_7 E_{hb_{ij}}(i,j) \quad (9)$$

$$E_{vdw} = \sum_{i,j} \sum_{ii,jj} w_8 E_{vdw}(i,j,ii,jj) \quad (10)$$

$$E_{\text{tor}} = \sum_i w_9 E_{\phi_i}(i) + w_9 E_{\psi_i}(i) \tag{11}$$

Note, the inter-residue θ and φ orientations are not symmetric, thus they must be summed over residues pairs *i*, *j* as well as the opposite direction *j*, *i*. Furthermore, the van der Waals potential also involves the interactions between each atom *ii* and *jj* from residues *i* and *j*. The detailed description of each energy term is described below.

$$E_{d_{ij}}(i,j) = \begin{cases} -\log\left(\frac{P(d_{ij}) + \epsilon}{P(d_{cut}) + \epsilon}\right), & d_{ij} < d_{cut} \\ 0, & d_{ij} \geq d_{cut} \end{cases} \tag{12}$$

where d_{ij} is the distance between two Cβ atoms for the Cβ distance restraints or two Cα atoms for the Cα distance restraints from residues *i* and *j*, $P(d_{ij})$ is the predicted probability by DeepPotential associated with the distance d_{ij}, and $P(d_{cut})$ is the probability for the final distance bin which corresponds to a distance between 19.5Å and 20Å. The pseudo count $\epsilon = 1E - 4$ is used to avoid issues when $P(d_{cut})$ is small. Cubic spline interpolation is used to interpolate between the energy at the different distance bins in order to make the potential differentiable for L-BFGS optimization.

$$E_{con_{ij}}(\mathrm{i,j}) = \begin{cases} -U_{ij}, & d_{ij} < 8\text{Å} \\ -\frac{1}{2}U_{ij}\left[1 - sin\left(\frac{d_{ij} - (\frac{8+D}{2})}{d_b}\pi\right)\right], & 8\text{Å} \leq d_{ij} < D \\ \frac{1}{2}U_{ij}\left[1 + sin\left(\frac{d_{ij} - (\frac{D+80}{2})}{(80-D)}\pi\right)\right], & D \leq d_{ij} \leq 80\text{Å} \\ U_{ij}, & d_{ij} > 80\text{Å} \end{cases} \tag{13}$$

where d_{ij} is the Cβ or Cα distance between the residue pair i and j. The depth of the potential, U_{ij}, is the predicted contact probability by DeepPotential. Overall, the potential is centered with a negative well at an 8 Å cutoff, with a strong force from 8 Å to D (=8 Å + d_b), followed by a weaker force from D to 80 Å, which is used to push the target residue pairs towards the well when they are far apart. Here, the gradient width (d_b) of the contact well is the only free parameter of the potential, which depends on the protein size and determines the convergence speed and satisfaction rate of the contact maps. As shown in Table S13, d_b is typically narrow, e.g., 6 Å, when the length of the target is relatively small, e.g. < 100 residues. On the other hand, the well width increases to 12 Å when the length is >200 amino acids, since residue pairs from larger proteins are more difficult to draw together, a wider well is used to draw the candidate residue pairs that are further apart in distance close together. It is important that the contact potential is designed in a way that the potential curve is continuous and smooth (with $\partial E/\partial d = 0$) at all three transition points of $d_{ij} = 8$, D and 80 Å, so that the contact restraints can guide the gradient-based folding simulations.

$$E_{\Omega_{ij}}(i,j) = \{-\log(P(\Omega_{ij}) + \epsilon) \tag{14}$$

$$E_{\theta_{ij}}(i,j) = \{-\log(P(\theta_{ij}) + \epsilon) \tag{15}$$

$$E_{\theta_{ji}}(j,i) = \{-\log(P(\theta_{ji}) + \epsilon) \tag{16}$$

$$E_{\varphi_{ij}}(i,j) = \{-\log(P(\varphi_{ij}) + \epsilon) \tag{17}$$

$$E_{\varphi_{ji}}(j,i) = \{-\log(P(\varphi_{ji}) + \epsilon) \tag{18}$$

where Ω_{ij}, θ_{ij}, and φ_{ij} are the inter-residue orientations predicted by DeepPotential between residues i and j defined in Figure S3. Furthermore, given that θ and φ are not symmetric for a residue pair, θ_{ji}, and φ_{ji} are the inter-residue orientations between residues j and i. The pseudo count $\epsilon = 1E-4$ is used to avoid issues when the predicted probability is small. Cubic spline interpolation is used to interpolate between the energy at the different orientation bins in order to make the potential differentiable for L-BFGS optimization.

$E_{hb}(i,j)$ was adapted from EvoEF[1] and is used to calculate the hydrogen-bonding interactions between potential hydrogen bond donor/acceptor pairs for atoms i and j, one of which should be a polar hydrogen. $E_{hb}(i,j)$ is a linear combination of three energy terms that depend on the hydrogen-acceptor distance (d_{ij}^{HA}), the angle between the donor atom, hydrogen and acceptor (θ_{ij}^{DHA}), and the angle between the hydrogen, acceptor and base atom (φ_{ij}^{HAB}):

$$E_{hb}(i,j) = w_{d_{HA}} E(d_{ij}^{HA}) + w_{\theta_{DHA}} E(\theta_{ij}^{DHA}) + w_{\varphi_{HAB}} E(\varphi_{ij}^{HAB}) \tag{19}$$

where:

$$\begin{cases} E(d_{ij}^{HA}) = \begin{cases} -\cos\left[\frac{\pi}{2}(d_{ij}^{HA} - 1.9)/(1.9 - d_{min})\right], d_{min} \leq d_{HA} \leq 1.9 \\ -0.5\cos[\pi(d_{ij}^{HA} - 1.9)/(d_{max} - 1.9)] - 0.5, \quad 1.9\text{ Å} < d_{HA} \leq d_{max} \\ 0 \quad , \quad otherwise \end{cases} \\ E(\theta_{ij}^{DHA}) = -\cos^4(\theta_{ij}^{DHA}), \quad \theta_{ij}^{DHA} \geq 90° \\ E(\varphi_{ij}^{HAB}) = -\cos^4(\varphi_{ij}^{HAB} - 150°), \quad \varphi_{ij}^{HAB} \geq 80° \end{cases} \tag{20}$$

$$E_{vdw}(i,j,ii,jj) = \begin{cases} (vdw(ii) + vdw(jj))^2 - {d_{ij,ii,jj}}^2, if\ d_{ij,ii,jj} < vdw(ii) + vdw(jj) \\ 0, \quad otherwise \end{cases} \tag{21}$$

Here, $E_{vdw}(i,j,ii,jj)$ is the van der Waals energy between atoms ii and jj from residues i and j, respectively, where $vdw(ii)$ and $vdw(jj)$ are the van der Waals radii of atoms ii and jj and $d_{ij,ii,jj}$ is the distance between atoms ii and jj from residues i and j, respectively. The atoms ii/jj that are accounted for are the backbone atoms (N, Cα, C, and O) and the Cβ atoms/side-chain centers of mass.

$$E_{\phi_i}(i) = 1 - \cos(\phi_i - \phi_{i,pred})\ and\ E_{\psi_i}(i) = 1 - \cos(\psi_i - \psi_{i,pred}) \tag{22}$$

$E_{\phi_i}(i)$ and $E_{\psi_i}(i)$ are the energy for the backbone torsion angles, where ϕ_i and ψ_i are the phi/psi torsion angles at residue i and $\phi_{i,pred}$ and $\psi_{i,pred}$ are the predicted torsion angles by Anglor[2].

Overall, the DeepFold force field consists of 24 weighting parameters, where the weights given to each of the deep learning restraints were separated into short ($|i-j| > 1$ and $|i-j| \leq 11$, where i is the residue index for residue i and j is the residue index for residue j), medium ($|i-j| > 11$ and $|i-j| \leq 23$) and long-range ($|i-j| > 23$) weights, which were determined by maximizing the TM-score on the training set of 257 non-redundant, Hard threading targets collected from the PDB that shared <30% sequence identity to the test proteins. Briefly, all the weights were initialized to 0, then the weight for each individual energy term was increased one-at-a-time and the DeepFold folding simulation were run using the new weights. Following this initial optimization, the weights were carefully fine-tuned by adjusting their values using a grid-searching technique around the optimized values.

References

1. Huang X, Pearce R, Zhang Y. EvoEF2: accurate and fast energy function for computational protein design. *Bioinformatics* **36**, 1135-1142 (2020).
2. Wu S, Zhang Y. ANGLOR: a composite machine-learning algorithm for protein backbone torsion angle prediction. *Plos One* **3**, e3400 (2008).
