# Supplementary material for: Fast and accurate Ab Initio Protein structure prediction using deep learning potentials
Source: PLoS Comput Biol. 2022 Sep 16;18(9):e1010539. doi: 10.1371/journal.pcbi.1010539 (PMC9518900; doi:10.1371/journal.pcbi.1010539)
Supplement: Attachment — Submitted filename: response_to_reviewers.pdf [file pcbi.1010539.s023.pdf]

Response to Reviewer #1

We very much appreciate the comments from the Reviewer, which were helpful for improving the quality of the manuscript. Specifically, the Reviewer raised a comment on the impact of sequence correlation and/or machine learning technique on the progress of protein structure prediction, for which we have partly rewritten the paragraph in the Introduction to integrate the idea and reference. Below, we include point-by-point replies to each of the Reviewer's comments, where all changes have been highlighted in yellow in the manuscript.

1. **The Reviewer commented:**

> ***The authors describe in this paper the important progress that they have made in protein structure predictions with their new DeepFold pipeline by including the predicted contacts, a deep learning-based potential and a knowledge-based statistical force field. Despite the huge publicity for Alphafold2, predicting protein structres remains an important research problem. One only has to inspect the Alphafold database to see the large number of extended segments being specified as being of low confidence.***

We appreciate the positive comments from the Reviewer on the work and agree that protein structure prediction remains an important research problem where progress may still be made.

2. **The Reviewer commented:**

> ***Second paragraph – 'use of deep learning techniques to predict spatial restraints' is misleading since it is primarily the data from sequence correlations that lead to these gains, rather than the machine learning itself. There are new gains that can be obtained from improving protein sequence matching itself (Proteins 2021, 89:671), which yields many more of the structure contacts.***

This is an interesting point. Indeed, the improved ability to identify more reliable and abundant sequence homologs leading to improved correlation analysis has played an important role in the improvement in protein structure prediction accuracy. This is especially true given the incorporation of large metagenomics sequence databases, which has enabled the detection of more sequence homologs than previously attainable. However, the primary gains are largely due to the advances in the deep learning architectures. For example, on the 221 benchmark proteins, each of the control methods were given identical MSAs, thus removing the bias in homologous sequence detection. We can see from the presented results that as the deep learning models became increasingly more advanced/accurate, the modeling performance improved. For instance, C-I-TASSER uses contacts predicted by ResPRE (https://zhanggroup.org/papers/2019_21.pdf), which utilizes 22 residual blocks to predict contact maps for a query sequence, and achieved the lowest average TM-score of the deep learning methods. Then DMPfold further extended the modeling accuracy using deep ResNets to predict the distance maps, while trRosetta and DeepPotential use deep ResNets to predict the combined distance and orientation maps, providing more detailed information on the pairwise positioning of each residue in a protein structure. Lastly, the most recent advances by AlphaFold2/RosettaFold use deep transformer networks to predict protein structures. Thus, starting from the same MSAs, the modeling performance of the various methods greatly varies depending on the deep learning techniques that were employed.

Moreover, we can see from the results in the CASP experiments that advances in deep learning have led to a significantly decreased dependency on the quality of the generated MSAs and thus the availability of information that can be extracted from sequence correlation. For instance, Fig. 5B of the paper https://zhanggroup.org/papers/2021_11.pdf plots the relationship between the contact prediction accuracy and the MSA Neff values from CASP11-14. From the figure it can be seen that in CASP11-12 there was a large dependency between the MSA Neff value and the contact prediction accuracy. This is mainly due to the employment of direct coupling analysis and shallow neural network methods used to predict contacts, which heavily depended on the information from sequence correlation. However, CASP13 and CASP14 saw the widespread use of deep ResNets for contact prediction and distance prediction, respectively, where the methods demonstrated a greatly decreased dependency on the quality of the generated MSAs and thus the reliability of the sequence correlation information.

Overall, we think both sequence correlation and machine learning are important components for the progress. We have thus added the following text to the introduction to address this point. Meanwhile, we have cited Proteins 2021, 89:671 to enhance the point (Page 3):

> Throughout the last few years, the use of deep learning techniques to predict spatial restraints from sequence and/or multiple sequence alignment (MSA) has dramatically improved the accuracy of *ab initio* structure prediction [4]. For example, in CASP11 and CASP12, predictors primarily used direct coupling analysis from MSA and shallow neural networks to predict contact maps, where the prediction accuracy largely relied on the identification of abundant sequence homologs in order to accurately predict contacts based on the information from correlated mutation patterns [5]. In the CASP13 experiment, however, the top-ranked server groups, Zhang-Server and QUARK, used contact maps predicted by deep convolutional residual networks (ResNets) [6] to guide the I-TASSER [7] and QUARK [8] folding simulations, respectively, which greatly improved the contact prediction and folding accuracies for the physics- and knowledge-based modeling approaches. This was especially apparent for targets that lacked homologous templates and high-quality MSAs [5]. There are also gains that can be obtained from improving protein sequence matching itself, which yields many more of the structure contacts [10].

3. <u>The Reviewer commented:</u>

> ***The pipeline was tested on 221 non-redundant protein domains taken from scope and FM targets form various CASPs. Results were compared against several other prediction approaches and clear gains were seen.***
> ***In the Author Summary should 'spare' be 'sparse' on next to last line?***

We thank the Reviewer for the detailed reading of the manuscript and have corrected the mistake.

Response to Reviewer #2

We thank the Reviewer for their comments, which we found very helpful for improving the quality of the manuscript. The main concerns from the Reviewer involved the absence of a comparison with AlphaFold2 and RosettaFold, as well as a call for non-parametric statistical analysis. Therefore, in the revised manuscript, we added an additional section to the results as well as Tables S10/S11 and Figs. S4/S5 to compare the method with AlphaFold2 and RosettaFold. Additionally, we included the results from the non-parametric analysis in Tables S5 and S7-11. Below, we reply point-by-point to the comments of the Reviewer, where all changes have been highlighted in yellow in the manuscript.

1. **The Reviewer commented:**

> ***The authors present DeepFold, a new method for protein structure prediction that uses L-BFGS down-hill minimization of distance and orientation constraints, formulated as an energy function. The constraints are derived from both MSAs and simple physical considerations. A similar approach is implicit in AlphaFold1. The authors carefully analyze the contribution of different constraints, and combinations thereof, to the shape of the energy function and prediction quality, as manifested by the TM-scores of the resulted models. They also provide visual inspection of a few interesting test cases. The new method outperforms several important alternatives in both accuracy and speed. Notably however, in this study DeepFold is not compared with AlphaFold2 and RoseTTAfold, which are freely available for testing.***
>
> ***The major take-home message of this study is that the larger is the number of reliable constraints, and the more diverse they are, that is distance vs. orientation and MSA-based vs. physics-based, the smoother is the energy surface and consequently the more accurate are the predictions.***

We appreciate the concise summary and positive comments from the Reviewer.

2. **The Reviewer commented:**

> ***Overall, the manuscript is clearly written and interesting, with minor issues raised below. However, its major weakness is the lack of comparisons to AlphaFold2 and RoseTTAfold, which are likely to outperform DeepFold. Obviously, this speculation may be wrong, and anyway performance is not the only criterion for the evaluation of a research paper, but the "elephant in the room" here is far too big to ignore.***

We agree that a comparison with AlphaFold2 and RosettaFold is warranted, particularly given the fact that DeepFold uses restraints from DeepPotential, which was developed before the most recent advances. We have added in the following section to the Results as well as Tables S10/S11 and Figs. S4/S5 to the SI in order to answer this question. The results showed that despite the overall better performance of AlphaFold2 and RosettaFold, DeepFold demonstrated advantage on some targets that have extremely few sequence homologies. When combined with RosettaFold restraints, the overall performance of DeepFold outperforms both end-to-end and pyRosetta protocols of the RosettaFold programs (Pages 7-8):

> **Comparison of DeepFold with the most recently developed methods: AlphaFold2 and RosettaFold**
>
> Since DeepFold uses restraints from DeepPotential, which was developed before the advances made by AlphaFold2 [26] in CASP14, it is also of interest to compare the results against the most

recent self-attention-based neural network methods, namely, AlphaFold2 and RosettaFold [27]. Thus, in Figs. S4A-C, we provide a head-to-head comparison of the DeepFold modeling results utilizing the restraints from DeepPotential with RosettaFold and AlphaFold2 on the 221 test proteins in terms of the model TM-scores, where the results are summarized in Table S10. Overall, the average TM-score of the RosettaFold end-to-end pipeline was 0.812 and the average TM-score of the Pyrosetta version was 0.838, which were higher than the results by DeepFold (TM-score=0.751) with *p*-values of 3.6E-10 and 8.0E-22, respectively. Similarly, the average TM-score of AlphaFold2 was 0.903, which was higher than DeepFold with a p-value of 1.4E-49. These results were expected given that the advances in deep self-attention neural networks and end-to-end training by AlphaFold2 and, subsequently, RosettaFold showed greatly improved modeling accuracy over previously introduced convolutional ResNet architectures, such as DeepPotential.

Notably, there were 7 targets for which DeepFold outperformed AlphaFold2. In Fig. S5, we illustrate two examples where DeepFold generated models that were significantly more accurate than AlphaFold2. The first example is from SCOPe protein d1a34a, for which DeepFold generated a model with a TM-score of 0.613, while AlphaFold2 generated a model with a TM-score of 0.242. For this target, DeepMSA2 was not able to identify any sequence homologs, resulting in an MSA composed of only the query sequence and an extremely low normalized Neff value of 0.08. Nevertheless, DeepPotential generated accurate restraints with an MAE of 1.10 Å for the top $2*L$ distances, resulting in a higher quality model than that produced by AlphaFold2. The second example is from SCOPe protein d1s2xa, for which DeepFold generated a model with a TM-score of 0.590, while AlphaFold2 generated a model with a TM-score of 0.369. Again, for this target, DeepMSA2 was only able to identify two sequence homologs, which resulted in a very low normalized Neff value of 0.15. Additionally, the DeepPotential restraints were fairly inaccurate with an MAE of 2.54 Å for the top $2*L$ distances and 59.29° for the $2*L$ Ω orientations. Interestingly, even though the orientation restraints were inaccurate, their inclusion greatly improved the modeling accuracy, as the model built using only the contact and distance restraints possessed a low TM-score of 0.268, while the model built using the full set of contact/distance and orientation restraints had a TM-score of 0.514. Moreover, the addition of the general knowledge-based energy function further improved the TM-score to 0.590. This suggests that even when inaccurate, the combination of various restraints with a general energy function may act synergistically to filter out inaccuracies in the predictions. It is also noteworthy that the two preceding examples were from proteins with few to no homologous sequences. In fact, if we consider the 5 proteins in the benchmark dataset with the least homologous sequence information (<3 sequence homologs) and normalized Neff values <0.20, DeepFold generated more accurate models than AlphaFold2 for 4 of these targets, where the average TM-score of DeepFold was 0.528 compared to 0.398 for AlphaFold2. This suggests that, while deep self-attention-based protein structure prediction approaches have demonstrated an improved ability to fold proteins with few sequence homologs, the performance on the most extreme cases with few sequence homologies remains to be improved.

Lastly, given the importance of the most recent advances in protein structure prediction, we sought to determine whether or not they could be incorporated into DeepFold to further improve its performance. To answer this question, we utilized the restraints taken from RosettaFold, including the Cβ distances and orientations, as well as the Cα distances/contacts and Cβ contacts from DeepPotential to guide the DeepFold simulations. The results of this analysis are depicted in Table S11 and Figs. S4D-F, which present head-to-head comparisons between DeepFold utilizing the combined restraints with RosettaFold and AlphaFold2 in terms of the model TM-scores on the 221 benchmark proteins. It was shown that with the combined RosettaFold and DeepPotential restraints, DeepFold achieved an average TM-score of 0.844, which was higher than that attained by the end-to-end (TM-score=0.812) and Pyrosetta (TM-score=0.838) versions of RosettaFold with *p*-values of 2.4E-11 and 1.2E-2, respectively. These data demonstrate that the DeepFold knowledge-based force field and DeepPotential contact and Cα distance restraints may improve the results obtained by RosettaFold. Additionally, it shows that DeepFold is a versatile platform that can be easily adapted for any future advances in state-of-the-art deep learning restraint predictors.

3. **The Reviewer commented:**

Minor issues:
1. The authors compare methods and sets of constraints by average values of performance measures (e.g., TM-score), and test the significance of differences of these values by Student's t-test. Averages and parametric tests maybe misleading when applied to distributions that are not normal like the ones shown in Figure 2. I believe that Instead (or in addition), the authors should use more robust, non-parametric, values and tests.

This a great suggestion. We have added in a comparison of the Medians as well as p-values calculated using non-parametric Wilcoxon signed rank tests whenever the results from the various methods are compared (Tables S5 and S7-11).

4. The Reviewer commented:

2. The authors mention "16 targets with Neff values less than 1 ". I guess this is a mistake. Even an orphan has a Neff value of 1.

We thank the Reviewer for raising this important point. In the study, we used the normalized Neff values, where $normalized\ Neff = Neff/\sqrt{L}$ (L is the protein length). We have added the details on the Neff value calculation in Text S1 (see below) and clarified that it is the normalized Neff values we analyzed wherever it is mentioned.

Text S1: Calculation of the MSA Neff value.
In order to quantify the quality of an MSA, we define the number of effective sequences (Neff) as follows:

$$Neff = \frac{1}{\sqrt{L}} \sum\nolimits_{n=1}^{N} \frac{1}{1 + \sum_{m=1,m\neq n}^{N} I[S_{m,n} \geq 0.8]}$$

where L is the length of a query protein, N is the number of sequences in the MSA, $S_{m,n}$ is the sequence identity between the m-th and n-th sequences, and $I[\ ]$ represents the Iverson bracket, which means $I[S_{m,n \geq 0.8}] = 1$ if $S_{m,n} \geq 0.8$ or 0 otherwise.

5. The Reviewer commented:

3. When mentioning the use of Potts models as features, the authors should refer to previous studies that did it. Further, I believe that readers would benefit from some discussion about the meaning of Potts models in this context. If space is limited, we could do without a formal description of a much more known algorithm such as L-BFGS.

We thank the Reviewer for the suggestion. We have added the following description in the Methods section **"Spatial restraint prediction by DeepPotential"**, along with references to previous studies in Refs. 36-39 (Page 12).

Here, a Potts model is a specific type of Markov Random Field (MRF) model that is widely used in protein structure prediction [36-39]. Briefly, an MRF is a graphical model that represents each column of an MSA as a node that describes the distribution of amino acids at a given position (Potts model field parameters), where the edges between nodes indicate the joint distributions of amino acids at each pair of positions. The 2D coupling parameters can then be determined from the edge weights, where residue pairs that exhibit correlated mutation patterns will possess greater edge weights, which can be used to infer positions that should be closer together in 3D space. This is

based off of the intuition that if two residues are in contact with each other, then when one residue mutates, the contacting residue should also mutate in order to preserve the interaction. In DeepPotential, CCMpred [39] is used to fit the Potts model.

Response to Reviewer #3

We thank the Reviewer for the comments and concerns they raised, which helped as improve the quality of our manuscript and programs. The main concerns from the Reviewer involved the need to highlight some interesting case studies where the competing methods outperformed DeepFold, an analysis of targets that were non-redundant to the training sets of the deep learning predictors, as well as issues with the stand-alone package/web server. Below, we reply point-by-point to the comments of the Reviewer, where all changes have been highlighted in yellow in the manuscript.

1. **The Reviewer commented:**

> ***In this work, Pearce and colleagues present DeepFold, a novel method to predict high-quality protein three dimensional structures using an ab initio approach which is guided by restraints originating from deep learning.***
>
> ***In my opinion, DeepFold constitutes a significant advance in the field, as the authors convincingly demonstrate the high quality of results obtained while maintaining running times reasonable. This performance speed-up is achieved by exploiting the wealth of structural restraints obtained from deep neural network processing of sequence-derived information, which enables the application of the very efficient L-BFGS algorithm in a smoothed energy landscape, leading to fast convergence.***
>
> ***The manuscript is well written and provides detailed benchmarking to relevant methods, suggesting that DeepFold can yield high quality results in reasonable time, thus making DeepFold a new important addition in the toolset of protein structure prediction.***
>
> ***Below are some specific comments (grouped by manuscript section), which I hope will be helpful to the authors. In addition, a few typos and potential passages needing clarifications are mentioned in the end of my review.***

We thank the Reviewer for their summary and positive comments on the manuscript and work. We have addressed the issues raised by the Reviewer in the following.

2. **The Reviewer commented:**

> ***# Specific comments***
> ***+ Results***
> ***- The authors clearly demonstrate (Fig. 4) that DeepFold outperforms competing methods in the compiled benchmark data. I would find interesting a discussion on the identities/properties of the few proteins where DeepFold performed worse than its 'competitors'. Were these inferior predictions due to poor MSAs (e.g. low Neff) or is there another reason that the authors could identify?***

We thank the Reviewer for the excellent suggestion. We have added the following paragraph in the section **"Comparison of DeepFold with other leading modeling methods"** as well as Fig. S2 to the SI in order to explore some of the examples where the competitors outperformed DeepFold (Page 6).

> Interestingly, there were two targets (d1ltrd and d1nova) for which I-TASSER and C-I-TASSER produced models that were significantly more accurate than DeepFold. To examine the reason for the discrepancy in performance, Fig. S2 depicts the models generated by I-TASSER, C-I-TASSER, and DeepFold superposed with the native structures along with the top templates used by I-TASSER

and C-I-TASSER for these proteins. For d1ltrd, despite the fact that it was a hard threading target, LOMETS was able to identify a reliable template from the PDB (1prtI) with a coverage of 92.6% and a TM-score of 0.553; thus, both I-TASSER and C-I-TASSER constructed accurate models with TM-scores of 0.663 and 0.637, respectively. Conversely for DeepFold, the generated MSA contained few homologous sequences with a normalized number of effective sequences (or Neff, defined in Text S1) of 0.42, resulting in inaccurate predicted restraints with an MAE of 2.60 Å for the top 2*L distances. This ultimately lead DeepFold to produce an inaccurate model with a TM-score of 0.326. Additionally, the contact precision for the top L/2 contacts used by C-I-TASSER was only 50.0%, which is largely why the C-I-TASSER model was worse than the I-TASSER model. Similarly, for d1nova, LOMETS was able to identify a reliable template (PDB ID 1hofC) with a coverage of 100% and a TM-score of 0.544, which resulted in accurate I-TASSER and C-I-TASSER models with TM-scores of 0.631 and 0.713 for the two methods, respectively. Again, for DeepFold, the generated MSA was shallow with a normalized Neff value of 9.40. Nevertheless, the predicted distance restraints were still accurate with an MAE of 0.90 Å for the top 2*L distances; however, the predicted orientations were inaccurate, particularly the Ω orientation, which had an MAE of 31.3° for the top 2*L restraints. This resulted in a model with a TM-score of 0.546, which still possessed a correct fold, but was less accurate than the models generated by I-TASSER and C-I-TASSER. Unlike the previous example, the C-I-TASSER model was more accurate than the I-TASSER model for d1nova as the predicted contacts were accurate with a precision of 98.7% for the top L/2 contacts. These two examples highlight that even with the advances in deep learning methods, template-based modeling still remains important, particularly given the reliance of deep learning techniques on the generated MSAs, which may be lower quality than the identified templates for numerous targets.

3. The Reviewer commented:

+ Methods and Usage
- I was unable to get results on time for my review using the online server. However, I trust that the server performs as advertised, as the authors provide an example input for submission and the corresponding output, which includes a predicted model for the example query sequence along with the intermediate results (i.e. predicted secondary structure and spatial restraints). In addition, the authors have made available a GitHub repository with the DeepFold source code accompanied with detailed documentation on how to install, setup and execute the DeepFold suite. Therefore, both regular and more experienced users will find it easy to get their hands on this new method.

We apologize that the Reviewer's job was not able to be returned in time. We have checked the error and found that for very large proteins (>1000 amino acids) the MSA generation step exceeded the wall-time that was allotted to the job. We have fixed this error and had the job run through smoothly.

4. The Reviewer commented:

- In the https://zhanggroup.org/DeepFold/README.md file it is mentioned that "Perl and java interpreters should be installed". It would be helpful if the minimum required versions for these interpreters are mentioned along with any non-standard packages/classes on which the provided code is depending.

We thank the Reviewer for the suggestion. We have added in information on the required versions as well as packages that must be installed.

5. The Reviewer commented:

- In the manuscript the authors mention the use of DeepMSA2 for deriving a multiple sequence alignment based on the query sequence. However, if I am not mistaken, the code provided in the repository corresponds to an initial version (DeepMSA). I would suggest the authors were given the opportunity to choose which version to install/use.

This is a great point. We have added in the option to download DeepMSA2.

6. <u>The Reviewer commented:</u>

- In page 13 (last paragraph) qMSA is described and it is mentioned to use "HHblits2 to search against the Uniclust30 database". However it is unclear from this passage which is the query used at this point. A reader needs to consult the respective supplementary figure (Fig S1), where it is shown that this step is executed using the original query.

We thank the Reviewer for this point. We have clarified that it is the original query sequence that is searched against the Uniclust30 database as follows (Page 12):

> The four remaining MSAs are generated using a procedure called quadruple MSA (qMSA in Fig. S1), which uses HHblits2 to search the original query sequence against the Uniclust30 database (version 2020_01) to create MSA-4.

7. <u>The Reviewer commented:</u>

In addition, it would be interesting to see the frequency by which each of the 7 types of MSA are chosen as the final MSA. Such information could provide additional insights for further speedup of the complete pipeline, since the MSA construction step requires significant computational resources at all levels - CPU, main memory, hard disk: potential users interested to install the code locally (especially in resource limited settings) would be interested in having information on which parts of this pipeline could be skipped without affecting quality of the results.

We thank the Reviewer for the suggestion. We have added the following section to the conclusion along with the histogram distribution of the number of times each MSA was selected to Fig. S6 (P11):

> This issue is particularly important for hard modeling targets, which often require extensive homologous sequence detection. As evidence of this, in Fig. S6, we plot the number of times each of the 7 MSAs produced by DeepMSA2 were selected for the 221 benchmark targets. From the figure, it can be seen that ~55% of the targets required searching beyond the MetaClust database, while only ~15% did not require searching through any metagenomics database.

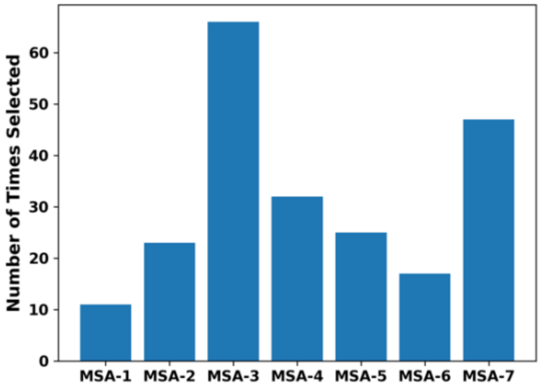

Figure S6. Histogram distribution of the number of times each of the 7 MSAs were selected by DeepMSA2 for the 221 benchmark targets. The MSA numbers correspond to those depicted in Fig. S1.

8. <u>The Reviewer commented:</u>

> ***- "DeepPotential models" were trained on a non-redundant set from the PDB. However, it is unclear by reading the respective section (pg. 14) whether the protein chains composing this training dataset exhibited any sequence similarity to the dataset used for benchmarking.***

This is a very important point. We have added the following paragraph to the **"Comparison of DeepFold with other leading modeling methods"** section as well as Table S8 to address this point (Page 7).

> It is of note that some of the proteins in the benchmark dataset may be homologous to the proteins that DeepFold and other methods were trained on, as these deep learning methods often require a comprehensive set of training proteins to properly generalize. Thus, in Table S8 we depict the results for different methods on the 90 proteins in the benchmark dataset that shared <30% sequence identity to any of the training proteins used by DeepPotential. From the table, it can be seen that the performances of each of the methods, including DeepFold, were quite similar to the results on the overall benchmark dataset, where the accuracy of each of the deep learning methods on the 90 proteins was only slightly lower (~0.7-2.8% lower average TM-scores) than their accuracy on the 221 benchmark targets, which is largely due to the lower Neff in MSA construction for the former dataset. Nevertheless, DeepFold still significantly outperformed each of the control methods on these targets.

9. <u>The Reviewer commented:</u>

> ***+ Typos/minor clarifications***
> ***- Page 9, paragraph 1, line 2: "inter-reside" should read "inter-residue"?***

We thank the Reviewer for the careful proofreading of the manuscript. We have fixed the typo.

10. <u>The Reviewer commented:</u>

> ***- Page Page 10, paragraph 2, line 4: I personally disagree with the use of the term "**highly** statistically significant".***

We changed “highly statistically significant” to “statistically significant”.

11. The Reviewer commented:

- Page 10, paragraph 3, line 3: Please provide a definition for Neff here, or give a pointer to the literature for readers not familiar with this term.

We have added the following description to Supplementary Text S1 to provide the details on the Neff calculation.

Text S1: Calculation of the MSA Neff value.

In order to quantify the quality of an MSA, we define the number of effective sequences (Neff) as follows:

$$Neff = \frac{1}{\sqrt{L}} \sum_{n=1}^{N} \frac{1}{1 + \sum_{m=1, m \neq n}^{N} I[S_{m,n} \geq 0.8]}$$

where L is the length of a query protein, N is the number of sequences in the MSA, Sm,n is the sequence identity between the m-th and n-th sequences, and $I[\ ]$ represents the Iverson bracket, which means $I[S_{m,n \geq 0.8}] = 1$ if $S_{m,n} \geq 0.8$ or 0 otherwise.

12. The Reviewer commented:

- Page 14: "DeepFold Force Field" parameter weights were initialized to zero values, then optimized. However, it is not mentioned what was the amount of increase to these weights (I wonder if the weights should be monotonically increased). The "grid-searching technique" also lacks implementation details.

We have added in the following description to the **“DeepFold Force Field”** section of the methods to explain the training procedure in more detail (Page 13).

Briefly, all the weights were initialized to 0, then the weight for each individual energy term was varied one-at-a-time by an increment of 0.25 in the range from [0, 25] and the DeepFold folding simulations were run using the new weights. The weight for each term that resulted in the highest average TM-score on the training set was accepted. After the initial weighting parameters were determined, 3 more optimization runs were carried out, where the weight for each energy term was again varied in a range from [0, 25] using an increment of 0.1 and the weighting parameters that resulted in the highest average TM-score on the training set were accepted. A final optimization run was carried out, where the weights were perturbed by [-2, 2] from their previously accepted values using an increment of 0.02 to precisely fine-tune their values.

13. The Reviewer commented:

- Page 19: In Fig 1, at the DeepPotential box, a label "2D input features" above the 2D residual blocks is probably missing.

Yes, we have added in the label “2D coupling features” to the figure. Thank you!
